# Synthesis and Evaluation of a Conjugate Vaccine Composed of *Staphylococcus aureus* Poly-N-Acetyl-Glucosamine and Clumping Factor A

**DOI:** 10.1371/journal.pone.0043813

**Published:** 2012-09-06

**Authors:** Tomás Maira-Litrán, Leticia V. Bentancor, Cagla Bozkurt-Guzel, Jennifer M. O'Malley, Colette Cywes-Bentley, Gerald B. Pier

**Affiliations:** Channing Laboratory, Department of Medicine, Brigham and Women's Hospital, Harvard Medical School, Boston, Massachusetts, United States of America; University of Padova Medical School, Italy

## Abstract

The increasing frequency, severity and antimicrobial resistance of *Staphylococcus aureus* infections has made the development of immunotherapies against this pathogen more urgent than ever. Previous immunization attempts using monovalent antigens resulted in at best partial levels of protection against *S. aureus* infection. We therefore reasoned that synthesizing a bivalent conjugate vaccine composed of two widely expressed antigens of *S. aureus* would result in additive/synergetic activities by antibodies to each vaccine component and/or in increased strain coverage. For this we used reductive amination, to covalently link the *S. aureus* antigens clumping factor A (ClfA) and deacetylated poly-N-β-(1–6)-acetyl-glucosamine (dPNAG). Mice immunized with 1, 5 or 10 µg of the dPNAG-ClfA conjugate responded in a dose-dependent manner with IgG to dPNAG and ClfA, whereas mice immunized with a mixture of ClfA and dPNAG developed significantly lower antibody titers to ClfA and no antibodies to PNAG. The dPNAG-ClfA vaccine was also highly immunogenic in rabbits, rhesus monkeys and a goat. Moreover, affinity-purified, antibodies to ClfA from dPNAG-ClfA immune serum blocked the binding of three *S. aureus* strains to immobilized fibrinogen. In an opsonophagocytic assay (OPKA) goat antibodies to dPNAG-ClfA vaccine, in the presence of complement and polymorphonuclear cells, killed *S. aureus* Newman and, to a lower extent, *S. aureus* Newman Δ*clfA*. A PNAG-negative isogenic mutant was not killed. Moreover, PNAG antigen fully inhibited the killing of *S. aureus* Newman by antisera to dPNAG-ClfA vaccine. Finally, mice passively vaccinated with goat antisera to dPNAG-ClfA or dPNAG-diphtheria toxoid conjugate had comparable levels of reductions of bacteria in the blood 2 h after infection with three different *S. aureus* strains as compared to mice given normal goat serum. In conclusion, ClfA is an immunogenic carrier protein that elicited anti-adhesive antibodies that fail to augment the OPK and protective activities of antibodies to the PNAG cell surface polysaccharide.

## Introduction

*Staphylococcus aureus* is a leading etiology of hospital-acquired infections worldwide. This versatile organism, including strains with a troublesome pattern of antibiotic resistance as accentuated by methicillin-resistant *S. aureus* (MRSA), cause a wide spectrum of diseases that range from mild skin and soft tissue infections to more severe invasive ones such as endocarditis, blood and lower respiratory tract infections, septic arthritis, osteomyelitis or deep-seated abscesses among others [1], [2]. With the emergence of these many difficult-to-treat strains the need to develop new antimicrobials and/or immunotherapeutic approaches to combat infections is more urgent than ever.

*S. aureus* elaborates a large collection of virulence factors including pore-forming toxins, superantigens, phagocytosis-evasion molecules and inhibitors of host immune effectors, as well as variably encoded and expressed microbial surface components recognizing adhesive matrix molecules (MSCRAMMs). Among these, a fair number have been tested individually as vaccine targets in preclinical studies using either active or passive immunization, including the capsular polysaccharides types 5 and 8 (CP5 and CP8), alpha-toxin (non-toxic derivative H35L), Panton-Valentine leukocidin (PVL), enterotoxins B, A, C1, nontoxic mutant toxic shock syndrome toxin 1 (TSST), lipoteichoic acid (LTA), and other virulence factors carrying the LPXTG motif needed for anchoring to the cell wall, such as fibronectin binding protein (FnBP), collagen binding protein (CnBP), clumping factor A (ClfA), and the iron surface determinant B protein (IsdB) [3]. Some vaccine antigens including CP5 and CP8 conjugate vaccines, the IsdB antigen, human polyclonal antibodies to ClfA and a humanized monoclonal antibody to LTA have reached phase III human trials. Despite promising results obtained in pre-clinical studies, all have failed to meet their defined endpoints in preventing *S. aureus* infection [3]. As a result of these disappointing outcomes with monovalent vaccine components, a shift towards use of polyvalent vaccines has garnered significant interest.

To asses if additive or synergistic protection could be engendered in a multivalent vaccine, we evaluated a conjugate vaccine composed of two highly conserved *S. aureus* surface antigens, poly-N-β-(1–6)-acetyl-glucosamine (PNAG) and ClfA by using the protection-inducing deacetylated glycoform of PNAG (dPNAG) [4] conjugated to ClfA as a vaccine candidate against *S. aureus* infections. Previous work carried out in our laboratory has already demonstrated the ability of a vaccine composed of dPNAG conjugated to the carrier protein diphtheria toxoid (dPNAG-DT) to induce high titers of opsonic and protective antibodies [4]. In addition to the potential additive/synergistic activity between antibodies to dPNAG and ClfA the synthesis of a bivalent dPNAG-CflA vaccine is also aimed to potentially expand the coverage of a single-component vaccine to include *S. aureus* strains expressing only one of the vaccine components.

Specifically this study investigated the value of ClfA as a carrier protein for dPNAG, the functional activity and specificity of antibodies to the dPNAG-ClfA vaccine using anti-adhesive and opsonic killing assays, and protective efficacy in a mouse model of *S. aureus* bloodstream infection.

## Materials and Methods

### Ethics Statement

This study was carried out in strict accordance with the recommendations in the Guide for the Care and Use of Laboratory Animals of the National Institutes of Health. All animal protocols were reviewed and approved by the Harvard Medical Area Standing Committee on Animals IACUC which has Animal Welfare Assurance of Compliance number A3431-01 on file with the Office of Laboratory Animal Welfare of the U.S. Public Health Service. During all animal experimentation procedures all efforts were made to minimize suffering.

Studies involving human subjects were approved by the Partners Health Care System Institutional Review Board (IRB). All subjects donating blood provided written informed consent to participate in the studies.

### Bacterial strains and growth conditions

The strains used in this work (Table 1) were routinely grown to stationary phase in tryptic soy broth (TSB) supplemented with 1% glucose. When necessary, TSB as well as tryptic soy agar (TSA) were supplemented with ampicillin (100 µg/ml), tetracycline (10 µg/ml) or erythromycin (10 µg/ml).

**Table 1:** Bacterial strains used in this study.

*S. aureus* strains	Description	Source
Newman	Serotype 5 strain	[19]
Newman Δ*ica*	Newman Δ*ica*::*tet*; Tet^R^	[20]
DU5852	Newman *clfA*::Tn*917*; Ery^R^	[21]
MN8	Serotype 8 strain	[22]
476	Non-typable strain	[23]

*E. coli* strains		
*E. coli* XL1-Blue pCF41-(221–559)	Derivative of expression plasmid pQE30 (Qiagen Inc.) containing codons for ClfA amino acids 221–559. Used for the purification of rClfA_221–559_ protein contains an N-terminal extension of six histidine residues; Amp^R^	[24]

### PCR detection of *icaABCD* and *clfA* genes in *S. aureus* strains

Primers pairs icaF (5′-CCAGAGAAATTAGATATTCATTGAACAAGAAGC-3′)/icaR (5′-CATGCCGACACCTATACATAATCCTAAAATGAA-3′) and ClfA-F (5′-CGGAAAAAATCGATTGGCGTGGCTT-3′)/ClfA-R (5′-GTAATGAACCTATTGATGCTAATAATCCCCA-3′) were used to amplify the entire *icaABCD* locus or the *clfA* gene, respectively, from genomic DNA extracted from the strains *S. aureus* Newman, 476 and MN8. The genomic DNA was isolated by using the Wizard genomic DNA purification kit (Promega) according to the manufacturer's instructions.

### Purification of clumping factor A

Recombinant clumping factor A (ClfA) protein, residues (221–559), containing an N-terminal extension of six histidine residues was expressed from the plasmid pCF41carried by *E. coli* XL-1 Blue and kindly provided by Dr Timothy Foster. *E. coli* was grown in LB supplemented with ampicillin in a 6-liter fermentor at 37°C until the optical density at 650 nm (OD_650 _nm) reached 1. The temperature was maintained at 37°C, isopropyl β-D-1-thiogalactopyranoside (IPTG) was added to a final concentration of 0.5 mM, and growth was continued for 3 h. Cells were pelleted (6,000 *g*×10 min at 4°C), resuspended in 30 ml of phosphate-buffered saline (PBS) with complete EDTA-free protease inhibitor cocktail (Roche Applied Science) and sonicated using 3 cycles of 20 s at 4 W (Sonic Dismembrator) followed by 10 s of rest in an ice-water bath. After cell debris was removed by centrifugation (6,000 *g*×10 min at 4°C), the supernatant was filtered and ClfA purified by affinity chromatography on a Ni_2_-chelate column (Novagen) following the manufacture's instructions. The protein was further purified by size exclusion chromatography (SEC) on Sephacryl S-200 (Amersham Biosciences) and endotoxin removed using Detoxi-Gel (Pierce, Rockford, IL).

### Coupling of dPNAG to ClfA

ClfA was coupled to purified dPNAG using the reductive amination reaction. Aldehyde groups were introduced onto the surface of ClfA by treating the protein with an excess of glutaraldehyde and the activated ClfA was subsequently reacted with dPNAG using the abundant free amino groups present on this polysaccharide in the presence of sodium cyanoborohydride (NaBH_3_CN) (Matreya, Pleasant Gap, PA).

#### Activation of ClfA with glutaraldehyde

ClfA was dissolved in 0.1 M carbonate buffer pH 10 at a concentration of 10 mg/ml and glutaraldehyde (Sigma) added to a final concentration of 1.25% v/v. This reaction was allowed to proceed for 2 h at room temperature and the gluraldehyde-activated protein dialyzed against PBS pH 7.4.

#### Coupling of glutaraldehyde-activated ClfA to dPNAG

dPNAG was first dissolved in 5M HCl, neutralized with an equal volume of 5 M NaOH and diluted in PBS pH 7.5 to a final concentration of approximately 5 mg/ml. This dPNAG solution was then mixed with an equal amount of gluraldehyde-activated ClfA in PBS and the pH adjusted to 7.5. Purified NaBH_3_CN was added to the mixture in a 20-fold excess over the amount of each of the components and the reaction allowed to proceed in the dark for 14 h at 37°C with mixing. After the conjugation reaction was completed the high molecular weight (MW) conjugate was purified from uncoupled dPNAG and ClfA by gel filtration chromatography on a Superose 6-prep-grade column (Amersham Biosciences). Fractions containing dPNAG-ClfA conjugate vaccine, which were identified on the basis of the earlier elution of both polysaccharide and protein compared with the elution of the non-conjugated components, were pooled, dialyzed against 20 mM HEPES buffer plus 50 mmM NaCl pH 8 and stored frozen at −20°C.

### Chemical analysis of dPNAG-ClfA conjugate vaccine

The dPNAG-ClfA conjugate vaccine was analyzed for its content of polysaccharide using hexosamine assay described by Smith and Gilkerson [5] with N-acetylglucosamine as the standard and for protein by the Bradford method [6] with bovine serum albumin (BSA) as the standard.

### Antiserum production

Antisera to purified dPNAG-ClfA were raised in various animal hosts including two rabbits, two rhesus monkeys and a goat. Antibodies to dPNAG-ClfA were raised in New Zealand White rabbits by subcutaneous (SC) immunization with two 10 µg doses of conjugated polysaccharide emulsified for the first dose in complete Freund's adjuvant and for the second dose in incomplete Freund's adjuvant, followed 1 week later by three intravenous (IV) injections of antigen in saline spaced 3 days apart. Rabbits were bled every 2 weeks and sera tested by the enzyme-linked immunosorbent assay (ELISA). Immune sera to dPNAG-ClfA was also raised in two rhesus monkeys by SC vaccination with a single dose of 100 µg of dPNAG-ClfA vaccine in aluminum hydroxide gel adjuvant (Alhydrogel 1.3*%*, Brenntag Biosector). Finally, a goat was immunized with the dPNAG-ClfA conjugate vaccine following the protocol described earlier for rabbits but with doses of 50 µg per injection in Freund's incomplete adjuvant. Serum samples used to compare the immunogenicity of the dPNAG-ClfA vaccine in rabbits, monkeys and a goat were collected four weeks after the last immunization.

### Immunogenicity of dPNAG-ClfA vaccine in mice

Groups of 10 mice (Swiss-Webster; female, 5–7 weeks of age) were immunized SC 3 times, 1 week apart, with 1, 5 or 10 µg of conjugated polysaccharide suspended in PBS. Blood was withdrawn weekly for a month and antibody titers to both dPNAG and ClfA determined by ELISA. Control groups received a mixture of unconjugated polysaccharide and protein in PBS in the same ratio as in the conjugate vaccine.

### ELISA

dPNAG and ClfA-specific antibodies were measured in sera obtained from mice, rabbits, monkeys and a goat by ELISA as described previously [7]. Purified dPNAG and ClfA were used to sensitize the ELISA plates at a concentration of 0.3 or 10 µg/ml respectively in phosphate buffer, pH 7.4.

### Phagocyte-dependent killing assays

Polymorphonuclear cells (PMNs) were prepared from fresh human blood collected from healthy adult volunteers as described earlier [4] under a protocol approved by the Institutional Review Board of Partner's Healthcare System and the concentration adjusted to 2.5×10^7^/ml in modified minimal essential medium (MEM)+1% BSA.

The complement source (1 ml of rabbit serum, Sigma) was adsorbed three times with *S. aureus* Newman at 4°C for 30 min in order to remove all non-specific antibodies present. After adsorption, the complement solution was centrifuged and filter sterilized.

The test sera for the OPK, goat antibodies to dPNAG-ClfA conjugate vaccine and normal goat sera (NGS), were first heated at 56°C for 30 min to inactivate endogenous complement activity and then absorbed three times at 4°C for 30 min with the PNAG-negative strain Newman Δ*ica* or the Δ*clfA* mutant DU5852 to remove antibodies not directed to the PNAG or ClfA antigens, respectively. The bacteria were then removed by centrifugation and the test sera filter sterilized.

Overnight cultures of the bacterial strains to be evaluated in OPKA were diluted 1∶100 in fresh TSB+1% glucose, grown to an OD_650 _nm of 0.4 (∼3×10^8^ colony forming units (CFU)/ml), and the final concentration adjusted to 4×10^6^ CFU/ml in MEM-1% BSA for use in the killing assay.

The actual phagocytic killing assay was performed by mixing 100 µl (each) of the PMNs suspension, target bacteria, dilutions of test sera, and the complement source. The reaction mixture was incubated on a rotor rack at 37°C for 90 min; samples were taken at time zero and after 90 min. A 10-fold dilution was made in TSB with 0.05% Tween to prevent bacterial aggregation, and samples were plated onto TSA plates. Tubes lacking any serum and tubes with NGS were used as controls, as were tubes containing serum and complement but lacking PMNs to control for potential aggregation of bacteria by the antibody, which would reduce the apparent CFU counts at the end of the assay. At the concentrations of antisera used in the opsonic killing assay, there was no reduction in CFU of >10% in samples lacking phagocytes but containing antibody and complement, indicating little agglutinating activity of the antibodies raised to dPNAG-ClfA vaccine. The percentage of killing was calculated by determining the ratio of the number of CFU surviving in the tubes with bacteria, leukocytes, complement, and goat anti-dPNAG-ClfA sera to the number of CFU surviving in tubes with the same components but containing NGA.

For inhibition studies, goat antiserum raised to dPNAG-ClfA was diluted 1∶10 and incubated for 90 min at 4°C with an equal volume of a solution containing 25 to 200 µg/ml of either purified dPNAG or ClfA. Subsequently, the antiserum was centrifuged, and the supernatant was used in the opsonophagocytic assay as described above.

### Murine bacteremia model

Groups of eight mice (FVB; female; 3–5 weeks of age) were immunized intraperitoneally (IP) with 0.4 ml of heat-inactivated (56°C for 30 min) immune goat sera raised to dPNAG-ClfA, dPNAG-DT (dPNAG conjugated to diphtheria toxoid protein) vaccines, or NGS 48 and 24 h before infection. Mice were challenged IV with the *S. aureus* strains Newman (4.3×10^7^ CFUs), MN8 (8.6×10^6^ CFUs), 476 (6.7×10^5^ CFUs), Newman Δ*ica* (7.5×10^7^ CFUs) or DU5852 (3.5×10^7^ CFUs), at the doses indicated, in 0.2 ml of PBS. Two hours later mice were sacrificed and samples of 0.5 ml blood obtained from the heart, mixed with 20 µl of heparin (Sigma), and plated onto TSA plates. Bacteremia was quantified by colony counts after overnight growth and values expressed as CFU/ml of blood.

### Preparation of affinity purified antibodies to ClfA

Three grams of CNBr-activated Sepharose 4B gel (Pharmacia Biotech, Uppsala, Sweden) was suspended in 250 ml of 1 mM HCl for 30 min at 4°C, and then the gel was washed with alternating amounts of 750 ml of 1 mM HCl, 100 ml of distilled water, and 300 ml of reaction buffer containing 0.1 M NaHCO_3_ and 0.5 M NaCl (pH 8.3). The gel was suspended in 10 ml of the reaction buffer (pH 8.3), incubated with 5 mg of ClfA overnight at 4°C, and then the coupled gel was packed into a low-pressure column and washed with 250 ml of 1 M glycine in reaction buffer (pH 8.3) to block unused activation sites. Subsequently, the coupled gel was washed three times with alternating 200 ml amounts of borate buffer containing 0.1 M boric acid and 1 M NaCl (pH 8.5), 100 ml of distilled water, 200 ml of acetate buffer containing 0.1 M sodium acetate and 1 M NaCl (pH 4.0), and 100 ml of distilled water and then was suspended in PBS.

Ten ml of rabbit antiserum raised to dPNAG-ClfA was passed through a CNBr-activated Sepharose 4B-packed affinity column that was immobilized with ClfA. After intensive washing with PBS, the specific anti-ClfA immunoglobulins, which bound to the beads were eluted with 0.1 M glycine-HCI, pH 2.7 buffer and collected into fractions that were immediately neutralized with 1 M Tris base buffer ammonium bicarbonate, pH 9 and monitored for their absorbance at OD_280 _nm. Fractions containing affinity-purified rabbit antibodies specific to ClfA were then pooled and quantified for protein content with the Bradford assay [6] using rabbit IgG as standart.

### Adherence of *S. aureus* to immobilized fibrinogen

Adherence of *S. aureus* to fibrinogen (Fn) (Sigma) was assessed in 96-well polystyrene plates coated for with 400 ng/well of Fn for 1 h at 37°C after which the remaining sites were blocked by with 200 µl of PBS 1% BSA for 1 h at 37°C.

The *S. aureus* strains Newman, MN8, 476 and the Newman Δ*clfA* derivative DU5852 were grown overnight on TSA plates, harvested by centrifugation, washed with PBS pH 7.2 and resuspended in PBS to an OD_650 _nm of 1.0. *S. aureus* cultures were then further concentrated 5 fold and pre-incubated with either rabbit affinity-purified antibodies to ClfA or control non-immune polyclonal rabbit IgG at concentrations ranging from 0.8–500 µg/ml in PBS for 1 h at room temperature. After Fn-coated plates were incubated with the *S. aureus* strains for 1 h at room temperature, wells were washed three times with PBS and bound cells fixed with 200 µl of glutaraldehyde (2% v/v in PBS) for 1 h and stained with crystal violet (Sigma) (0.5% v/v) for 5 minutes. Plates were rinsed with water, air-dried and the absorbance at 595 nm (OD_595 _nm) determined using an ELISA plate reader (BioTek Instruments, Winoski, Ill).

### Confocal laser scanning microscopy

Confocal laser scanning microscopy (CLSM) was used to evaluate the production of PNAG and ClfA in three *S. aureus* strains, Newman, MN8 and 476. *S. aureus* strains were grown overnight at 37°C in TSB supplemented with 1% glucose, washed twice with PBS and blocked overnight with PBS 1% BSA and 10% normal rabbit sera (NRS). Cells were washed twice with PBS and a 10 µl aliquot of the cell suspension was air-dried onto a glass slide. Bacteria were fixed to the slide with methanol for 1 min at room temperature. At this point *S. aureus* samples were incubated for 2 h at room temperature with antibodies to PNAG (human monoclonal antibody (mAb) F598 conjugated to Alexa Fluor 488) and ClfA (goat dPNAG-CflA sera that had been previously absorbed four times at 4°C with the ClfA-negative *S. aureus* strain DU5852) in PBS with 0.5% BSA and 10% NRS. As controls we included *S. aureus* samples labeled with a human alginate-specific mAb F429 conjugated to Alexa Fluor 488 and NGS that had been previously absorbed with *S. aureus* DU5852. Samples were washed three times with PBS and further incubated for 1 h at room temperature with a secondary donkey anti-goat IgG conjugated to Alexa Fluor 568 (Invitrogen) diluted 1∶250 in PBS 0.5% BSA plus the nucleic acid stain Syto 62, (Invitrogen) at a final concentration of 5 µM. Samples were washed twice with PBS, and mounted with Mowiol mounting media and a glass coverslip and observed with a Zeiss LSM 5 Pascal confocal inverted microscope equipped with an Argon 488 nm laser, a HeNe1 543 nm laser, and a HeNe2 633 nm laser. Samples were viewed with a Plan Apochromat 63×/1.4 oil objective and data analyzed with Zeiss LSM Imaging software.

### Statistical analysis

All statistical analyses were performed with Prism 4.0 (GraphPad Software, http://www.graphpad.com/). An unpaired *t* test was used to compare the IgG antibody titers generated against ClfA between mice immunized with the conjugate dPNAG-ClfA vaccine and the mixture of unconjugated dPNAG+ClfA. One-way analysis of variance (ANOVA) was applied for comparisons of the opsonophagocytic killing among the isogenic *S. aureus* Newman strains and the protective efficacy of dPNAG-DT and dPNAG-ClfA antibodies versus NGS in bacteremia studies followed by Tukey's post-hoc test. A *P*-value of <0.05 was considered significant.

## Results

### Synthesis of dPNAG-ClfA conjugate vaccine

**Figure 1:**
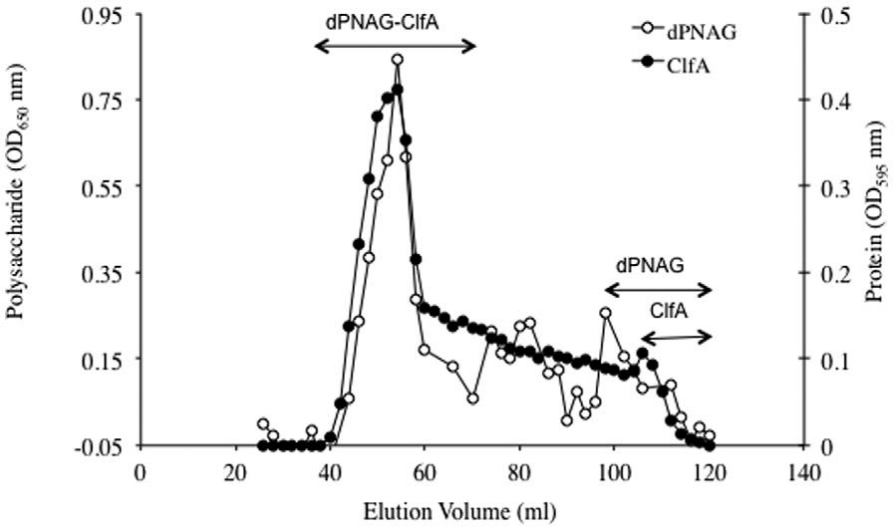
Size exclusion chromatography profile of dPNAG conjugated to ClfA through a Superose 6 gel column. Fractions were assayed for polysaccharide by the hexosamine assay (OD_650 _nm) and for protein with the Bradford assay (OD_595 _nm). Double-headed arrows indicate the fractions containing conjugated dPNAG and ClfA, as well as where the unconjugated polysaccharide (dPNAG) and protein (ClfA) eluted.

ClfA and dPNAG were covalently linked via reductive amination in the presence of NaBH_3_CN. Results presented in Figure 1 show that both ClfA (OD_595 _nm) and dPNAG (OD_650 _nm) co-eluted in the void volume of the column (void volume: approximately 40 ml) and significantly earlier than the unconjugated dPNAG and ClfA that eluted at approximately 100 and 110 ml, respectively, denoting the formation of a large MW dPNAG-ClfA conjugate. To identify fractions containing dPNAG-ClfA conjugate free of unconjugated ClfA and/or dPNAG all fractions were analyzed by sodium dodecyl sulphate-polyacrylamide gel electrophoresis (SDS-PAGE) followed by silver staining. Fractions in the elution volume of 42–70 ml contained high MW dPNAG-ClfA conjugate which did not enter the SDS-PAGE gel (no signal detected by silver staining) whereas the remaining fractions contained various levels of free dPNAG and/or ClfA and were not included in the final conjugate vaccine pool (data not shown).

Pooled fractions containing the purified dPNAG-ClfA conjugate were dialyzed overnight against HEPES buffer pH 8 and analyzed for polysaccharide and protein content using the Gilkerson-Smith and Bradford assays, respectively. The dPNAG-ClfA conjugate vaccine contained 31% dPNAG and 69% ClfA.

### Immunogenicity of dPNAG-ClfA conjugate vaccine in various animal species

**Figure 2:**
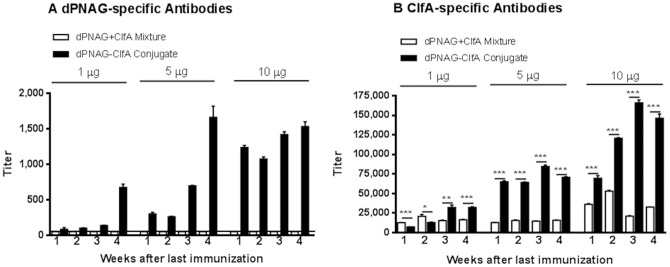
Immunogenicity of dPNAG-ClfA vaccine in mice. Mean titers of IgG antibodies to PNAG and ClfA in sera from mice (n = 10) immunized three times at weekly intervals with 1, 5 or 10 µg of dPNAG−ClfA conjugate vaccine or dPNAG mixed with ClfA (dPNAG+ClfA). Sera were collected weekly for 4 weeks starting 1 week after the last immunization and were tested by ELISA for antibodies to dPNAG and ClfA. A) IgG antibody levels to dPNAG in sera of mice vaccinated with dPNAG−ClfA conjugate vaccine or a mixture of unconjugated dPNAG+ClfA. Horizontal line depicts the limit of detection (titer = 25). For clarity, titers from mice receiving the mixture dPNAG+ClfA, which were below the limit of detection, were assigned a value of the limit of detection. B) IgG antibody titers to ClfA in sera of mice given the dPNAG−ClfA conjugate vaccine or control dPNAG+ClfA. Bars represent means and error bars indicate standard deviations. *P* as determined by unpaired *t* test. * *P*<0.05, ** *P*<0.005 and *** *P*<0.0001. All pre-immune titers were <25.

Mice (n = 10) were immunized three times with 1, 5 or 10 µg of either the dPNAG-ClfA conjugate or a mix of comparable amounts of unconjugated dPNAG plus ClfA (dPNAG+ClfA) as a control. As seen in Fig. 2A mice vaccinated with the dPNAG-ClfA conjugate responded with IgG antibodies to dPNAG in a dose-dependent manner. No antibodies to dPNAG were detected in the mice receiving the unconjugated dPNAG and ClfA mixture (Titer below level of detection of 25) (Fig. 2A). Similarly, mice vaccinated with the dPNAG-ClfA conjugate developed dose-dependent antibody titers to ClfA while the animals given the dPNAG+ClfA mix responded with significantly lower antibodies to ClfA (Antibody titers to ClfA in dPNAG-ClfA conjugate versus dPNAG+ClfA mixture ***P<0.0001 unpaired *t* test in all three doses and time points post-immunization except for mice immunized with 1 µg at week 2 and 3 post immunization that were *P<0.05 and **P<0.005, respectively) (Fig. 2B).

The immunogenicity of dPNAG-ClfA was also evaluated in two rabbits, two rhesus monkeys and a goat. As shown in Table 2 the dPNAG-ClfA vaccine was highly immunogenic in all three of these animal species, eliciting high antibody titers to both dPNAG and ClfA antigens. Interestingly there were differences in the levels and the relative response to each vaccine component among the three animal hosts. These differences might be explained in terms of doses, immunization regimes and/or host-specific differences among the various animal hosts.

**Table 2:** IgG titers to dPNAG and ClfA in various animal host species including two rabbits, two rhesus monkeys and a goat vaccinated with dPNAG−ClfA.

Coating Antigen	Rabbit 1	Rabbit 2	Monkey 1	Monkey 2	Goat
dPNAG	1,330,000	1,220,000	10,858	26,389	506,433
ClfA	102,000	107,000	730	719	1,118,184

Serum samples from all animal hosts, collected four weeks after the last immunization, were measured by ELISA for antibody titers specific to dPNAG and ClfA. Titers determined by end-point linear regression.

### Characterization of PNAG and ClfA production in *S. aureus* Newman, MN8 and 476 strains

PCR amplification of the *icaABCD* locus and *cflA* genes from genomic DNA extracted from three representative *S. aureus* strains Newman (CP5), MN8 (CP8) and 476 (non-typable) resulted in amplification bands with sizes consistent of those of the *icaABCD* (3,415 bp) and *clfA* (2,802 bp) genes of *S. aureus* (data not shown).

**Figure 3:**
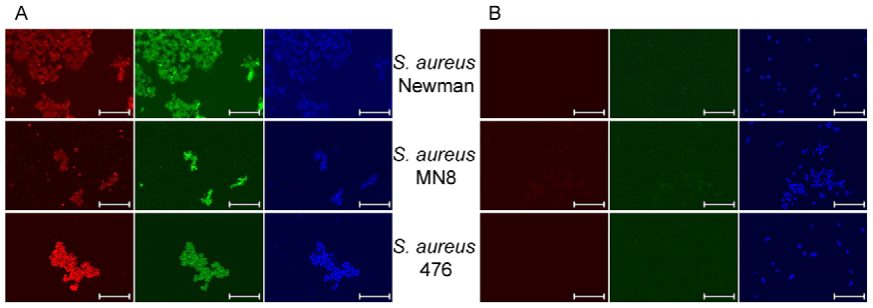
CLSM imaging of ClfA and PNAG expression by *S. aureus* Newman, MN8 or 476 strains. A) *S. aureus* labeled with mAb F598 to PNAG directly conjugated to Alexa Fluor 488 (F598-AF488), goat antibody to dPNAG−ClfA conjugate vaccine that had been previously absorbed with *S. aureus* Newman DU 5852 to remove antibody to dPNAG and a secondary donkey antibody to goat IgG conjugated to AF568 and the nucleic acid-specific fluorescent dye Syto 62. B) *S. aureus* stained with mAb F429 to *P. aeruginosa* alginate directly conjugated to AF488, NGS and a secondary donkey antibody to goat IgG conjugated to AF568 and Syto 62. For both panels A and B, the first, second and third columns represent *S. aureus* samples viewed in the red (ClfA), green (PNAG) and blue (bacterial DNA) channels, respectively. Bar = 10 µm.

In addition to PCR we conducted confocal laser scanning microscopy (CLSM) studies to investigate the production of PNAG and ClfA by these three *S. aureus* strains. For these experiments PNAG was labeled with the human IgG1 mAb F598 specific to PNAG directly conjugated to Alexa fluor 488 (F598-AF488) and ClfA was labeled with goat antibodies raised to the dPNAG-ClfA antibodies that had been previously absorbed with *S. aureus* Newman DU5852 (Δ*clfA*) to remove all antibodies raised to dPNAG followed by a secondary donkey anti-goat IgG AF568. As controls, bacterial samples were stained with a human IgG1 mAb to *Pseudomonas aeruginosa* alginate, mAb F429, also directly conjugated to AF488 and with non-immune goat serum. As presented in Figure 3A (first and second columns) there were high levels of ClfA (red channel) and PNAG (green channel) antigens detected by specific antibody binding in all three *S. aureus* strains. Conversely, *S. aureus* staining with control mAb F429-AF488 and NGS resulted in almost undetectable levels of red or green fluorescent labels, (Fig. 3B first and second columns, respectively). Labeling with the nucleic acid-specific Syto 62 fluorescent dye was also included in the experiments and results shown in the third columns of both panels.

### Inhibition of *S. aureus* binding to fibrinogen by antibodies to ClfA

**Figure 4:**
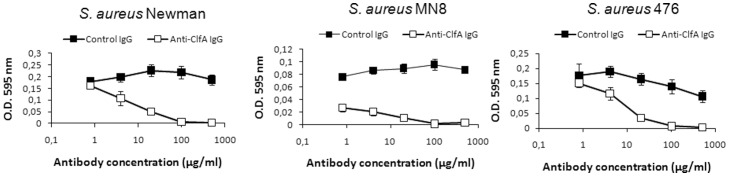
Antibody to ClfA inhibits *S. aureus* binding to immobilized fibrinogen. Affinity-purified rabbit anti-ClfA IgG's (□) and control non-immune rabbit IgG's (▪) were tested at various concentrations (0.8 to 500 µg/ml) for the their ability to inhibit the binding of *S. aureus* Newman, MN8 or 476 strains to fibrinogen. Fibrinogen-coated plates were incubated with *S. aureus* for 1 h, fixed with glutaraldehyde for 1 h and stained with crystal violet (0.5% v/v) for 5 minutes. After plates were air-dried the absorbance at 595 nm (OD_595 _nm) was determined using an ELISA plate reader. Data points represent the average of three independent experiments ± SEM.

Affinity purified antibodies specific to ClfA or control rabbit IgG were incubated at concentrations ranging from 0.8 to 500 µg/ml with *S. aureus* bacteria which were then added to Fn-coated microtiter plates. Bacterial binding was detected by crystal violet staining and quantification at OD_595 _nm. As shown in Figure 4 affinity-purified antibodies to ClfA were highly effective at blocking the binding of the three *S. aureus* strains Newman, MN8 and 476 to immobilized Fn in a dose-dependent manner. In addition, there was no detectable binding of the ClfA-negative strain *S. aureus* DU5852 to immobilized Fn (data not shown).

### Phagocyte-dependent killing by goat antibodies to the dPNAG-ClfA conjugate vaccine

**Figure 5:**
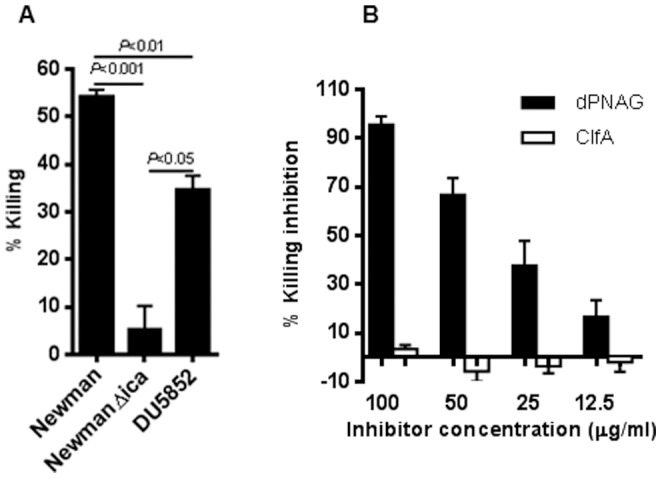
Opsonophagocytic killing activity and specificity of goat sera raised to dPNAG−ClfA conjugate vaccine. A) Opsonic killing of *S. aureus* Newman, Δ*ica* and DU 5852 (Δ*clfA*), by a 1∶10 dilution of goat antiserum raised to dPNAG−ClfA in the presence of polymorphonuclear cells and complement. Bars represent mean percentages of killing ± SEM. *P* as determined by one-way ANOVA with Tukey's post-hoc analysis. B) Inhibitory capacity of dPNAG and ClfA antigens in the opsonophagocytic assay. Inhibition of opsonic killing of *S. aureus* Newman by the goat serum raised to dPNAG−ClfA conjugate vaccine with purified dPNAG (solid bars) or ClfA (open bars) antigens at the indicated concentration. Bars represent means of three independent experiments ± SEM.

As shown in Figure 5A, antisera to dPNAG-ClfA at a 1∶10 dilution promoted the killing of wild type *S. aureus* Newman (54% killing) but there was no killing of the PNAG-negative strain Newman Δ*ica* (5.4% killing) (Killing of *S. aureus* Newman versus Δ*ica*; *P*<0.001, one-way ANOVA). This antiserum also killed the ClfA-negative mutant DU5852 but to a lower extent (35% killing) (Killing of *S. aureus* Newman versus DU5852; *P*<0.01, one-way ANOVA). This reduction in susceptibility of the ClfA-negative strain DU5852 in comparison to wild type *S. aureus* Newman is most likely attributable to the variation in PNAG retention on the bacterial cell surface when other factors are changed, as retention is often due to post-synthetic effects such as that of the extracellular IcaB deacetylase [8], [9]. Based on our previous experience in testing mAbs and polyclonal sera to dPNAG in the OPKA we found that opsonic killing levels >30% identify antisera with *in vivo* protective activity [4], [10].

To ascertain the specificity of the OPKA, purified dPNAG or ClfA were added to diluted antisera then tested for killing activity. Purified dPNAG inhibited the OPKA of goat antisera to dPNAG-ClfA conjugate, with inhibition titering out in a dose-dependent manner (Fig. 5B). ClfA failed to inhibit the opsonic killing of *S. aureus* Newman by antibodies elicited by the dPNAG-ClfA conjugate at all concentrations tested, indicating that the OPKA measured here was all due to antibody to PNAG.

### Protective efficacy of goat antiserum to the dPNAG-ClfA conjugate vaccine in a murine bacteremia model

**Figure 6:**
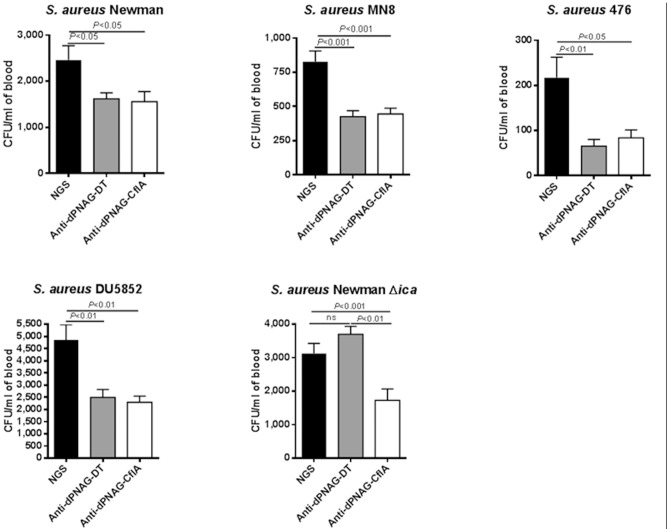
Protective efficacy of dPNAG−ClfA vaccine in a murine bloodstream infection model. Comparative protective efficacy elicited by goat immune sera raised to dPNAG−ClfA and dPNAG-DT conjugate versus NGS against *S. aureus* strains Newman, MN8, 476, Newman Δ*ica*, or DU5852 in a murine bacteremia model. Groups of eight FVB mice were immunized IP with 0.4 ml of heat-inactivated antisera raised to dPNAG-DT, dPNAG−ClfA vaccines or with control NGS, 48 and 24 h before IV challenge with the *S. aureus* strains Newman (4.3×10^7^ CFUs), MN8 (8.6×10^6^ CFUs), 476 (6.7×10^5^ CFUs), Newman Δ*ica* (7.5×10^7^ CFUs), or DU5852 (3.5×10^7^ CFUs), sacrificed 2 h post infection and the number of CFU/ml of blood estimated by serial dilutions and plating. Bars indicate the mean CFU per ml of blood, error bars the SEM. *P* values as determined by one-way ANOVA with Tukey's post-hoc analysis. (ns: not significant).

We evaluated the ability of goat antisera to the dPNAG-ClfA conjugate vaccine along with goat antisera raised to a conjugate of dPNAG and diphtheria toxoid (dPNAG-DT) that had comparable antibody titers to PNAG, to reduce bacterial levels in the blood of mice 2 h after IV injection. As shown in Figure 6, mice injected with antibodies to the dPNAG-ClfA conjugate vaccine had a significant reduction in blood CFU levels of *S. aureus* Newman 2 h post infection in comparison to animals given control NGS. A comparable reduction in the blood levels of *S. aureus* Newman was achieved with the antiserum to the dPNAG-DT conjugate vaccine, suggesting there was an immeasurable contribution of the antibodies to ClfA to the protective efficacy of the antibodies to the dPNAG-ClfA vaccine. Comparable results were also seen with the *S. aureus* strains MN8 and 476 (Fig. 6).

When similar protection studies were carried out with *S. aureus* DU5852 which does not elaborate ClfA, mice immunized with dPNAG-DT or dPNAG-ClfA had significantly and comparable lower bacterial blood levels than animals given non-immune NGS (dPNAG-DT and dPNAG-ClfA vs NGS *P*<0.01). On the other hand mice that had been passively immunized with antisera raised to the dPNAG-DT vaccine then infected with the PNAG-deficient strain Newman Δ*ica* had no significant changes in blood CFU levels in comparison to the control NGS (dPNAG-DT vs NGS; ns). However, mice infected with the Δ*ica* strain after passive immunization with antibodies raised to the dPNAG-ClfA vaccine exhibited significant bacterial blood clearance in comparison to the NGS-immunized group (dPNAG-ClfA vs NGS, *P*<0.01). These findings indicate that in the presence of the PNAG antigen, antibodies to ClfA are not effective at mediating protection from bacteremia. However in the absence of PNAG, the ClfA antigen could be either significantly more expressed or exposed on the surface of bacteria, allowing the antibody to ClfA to be protective in the bacteremia model of infection. Presumably this is by a non-OPK mechanism as antibody to ClfA does not appear to have significant opsonic activity against wild type or Δ*ica S. aureus* strains.

## Discussion

In this work we report the synthesis of a conjugate vaccine covalently linking two *S. aureus* surface components, ClfA and the deacetylated derivative of PNAG, and showed that immunization of mice with this conjugate vaccine dramatically enhanced the immunogenicity of both components of the vaccine.

The increased immunogenicity of ClfA in dPNAG-ClfA-vaccinated mice in comparison to animals receiving dPNAG plus ClfA may be explained in part by the likely enhenced immunogenicity of the high molecular weight, highly cross-linked, three-dimensional structure of the dPNAG-ClfA conjugate in comparison to the mixture of unconjugated ClfA and dPNAG.

On the other hand, the lack of immunogenicity of unconjugated dPNAG in mice immunized with 1, 5 or 10 µg doses of a mixture of dPNAG-ClfA was not entirely surprising. Previous studies carried out in our laboratory have demonstrated that mice immunized with 100 µg doses of the fully acetylated PNAG, which was used to prepare the dPNAG for the present study, failed to elicit a detectable IgG response [7].

The dPNAG-ClfA vaccine was also highly immunogenic in multiple animal species including rabbits, rhesus monkeys and a goat wherein vaccination elicited high antibody titers to both dPNAG and ClfA. Although the total number of animals immunized was limited to two rabbits, two monkeys and a goat we found relative differences in antibody responses to dPNAG and ClfA among the different animal hosts. These could relate to differences in vaccine dosage (10, 50 and 100 µg doses for rabbits, goat and monkeys respectively), immunization protocol (2 SC injections followed by 3 IV doses in rabbits and goat versus one single SC dose for the monkeys), type of adjuvant (Freund's incomplete for rabbits and the goat or alum for monkeys), used and/or to host-specific responses.

The goat antiserum was found to mediate opsonic killing of *S. aureus* that was specific to the dPNAG antigen. In addition, we found that affinity-purified antibodies to ClfA inhibited the adherence of three *S. aureus* strains to immobilized Fn *in vitro*. However, antibody to ClfA had negligible opsonic activity against *S. aureus* Newman and did not augment the protective efficacy of dPNAG-specific sera in a murine model of bacteremia against three PNAG- and ClfA-positive strains of *S. aureus*

Previous reports have demonstrated that the use of single vaccine antigens such as CP or ClfA resulted in protection of animals against *S. aureus* infections [11]–[14] but single component vaccines have failed in all human trials to date [3]. We therefore reasoned that the combination of PNAG and ClfA in the form of a bivalent dPNAG-ClfA conjugate vaccine might be required to confer full protection against *S. aureus* infection and/or to broaden the vaccine coverage to include *S. aureus* strains which do not fabricate either PNAG or ClfA. Moreover since *S. aureus* capsular polysaccharide have been shown to mask clumping factor A-mediated adherence of *S. aureus* to fibrinogen and platelets [15] then the addition of PNAG to ClfA-based vaccines might be advantageous.

The evidence presented in this work is in full agreement with previous reports showing that either polyclonal or monoclonal antibodies against ClfA inhibit the binding of *S. aureus* to immobilized fibrinogen *in vitro* [11], [16], [17], thought to be one correlate of protective efficacy. However, the sera raised to the dPNAG-ClfA conjugate was no better than antibodies raised to dPNAG conjugated to DT at reducing blood levels of *S. aureus* following IV challenge with three strains. These findings are consistent with a previous report showing that passive immunization of mice with affinity-purified ClfA specific antibodies could not prolong the survival in a sepsis model after receiving an intravenous lethal challenge of *S. aureus* N315 or *S. aureus* MW2 [18].

In conclusion the conjugation of dPNAG to ClfA, enhanced the immunogenicity of each component of the vaccine and elicited functional anti-adhesive and opsonic/protective antibodies specific to ClfA and dPNAG, respectively. However the lack of opsonic activity of antibodies to ClfA against wild type *S. aureus* Newman combined with their negligible protective efficacy against wild type *S. aureus* strains expressing PNAG suggests no major role for immunity to ClfA in *S. aureus* bacteremia. While levels of the PNAG-defective mutant of *S. aureus* Newman were reduced in the blood of mice passively administered the antibody to dPNAG-ClfA, this situation is not representative of human infections which occur almost exclusively with PNAG-producing *S. aureus*. Antibody to ClfA might be useful in other settings of *S. aureus* infections but incorporation of this bacterial component into a vaccine is made difficult by the poor availability of the antigen to antibodies when surface capsules, including both CP5 or CP8 and PNAG are made [15]. Coupled with the failure of the clinical trial using ClfA-enriched human immune globulins for prevention of *S. aureus* infections in neonates it appears that validating use of either passive antibody to this antigen or inclusion of ClfA in a vaccine will require better definition of where, when and how immunity to ClfA could augment human resistance to *S. aureus* infection.
